# “It’s like asking for a necktie when you don’t have underwear”: Discourses on patient rights in southern Karnataka, India

**DOI:** 10.1186/s12939-023-01850-5

**Published:** 2023-03-15

**Authors:** Meena Putturaj, Sara Van Belle, Anja Krumeich, Prashanth NS, Nora Engel

**Affiliations:** 1 Institute of Tropical Medicine, Antwerpen, Belgium; 2 Maastricht University, Maastricht, Netherlands; 3 Institute of Public Health, Bengaluru, India; 4 The University of Transdisciplinary Health Sciences and Technology, Bengaluru, India

**Keywords:** Patient rights, Critical discourse analysis, Health facilities, Human rights

## Abstract

**Background:**

Ensuring patient rights is an extension of applying human rights principles to health care. A critical examination of how the notion of patient rights is perceived and enacted by various actors through critical discourse analysis (CDA) can help understand the impediments to its realization in practice.

**Methods:**

We studied the discourses and discursive practices on patient rights in subnational policies and in ten health facilities in southern Karnataka, India. We conducted interviews (78), focus group discussions (3) with care-seeking individuals, care-providers, health care administrators and public health officials. We also conducted participant observation in selected health facilities and examined subnational policy documents of Karnataka pertaining to patient rights. We analyzed the qualitative data for major and minor themes.

**Results:**

Patient rights discourses were not based upon human rights notions. In the context of neoliberalism, they were predominantly embedded within the logic of quality of care, economic, and consumerist perspectives. Relatively powerful actors such as care-providers and health facility administrators used a panoply of discursive strategies such as emphasizing alternate discourses and controlling discursive resources to suppress the promotion of patient rights among care-seeking individuals in health facilities. As a result, the capacity of care-seeking individuals to know and claim patient rights was restricted. With neoliberal health policies promoting austerity measures on public health care system and weak implementation of health care regulations, patient rights discourses remained subdued in health facilities in Karnataka, India.

**Conclusions:**

The empirical findings on the local expression of patient rights in the discourses allowed for theoretical insights on the translation of conceptual understandings of patient rights to practice in the everyday lives of health system actors and care-seeking individuals. The CDA approach was helpful to identify the problematic aspects of discourses and discursive practices on patient rights where health facility administrators and care-providers wielded power to oppress care-seeking individuals. From the practical point of view, the study demonstrated the limitations of care-seeking individuals in the discursive realms to assert their agency as practitioners of (patient) rights in health facilities.

**Supplementary Information:**

The online version contains supplementary material available at 10.1186/s12939-023-01850-5.

## Background

Ensuring patient rights is an extension of applying human rights principles to health care [1]. Human rights aim to empower citizens and balance their power in relation to their state. Similarly, patient rights intend to empower patients, balance power, and prevent exploitation by physicians, other health workers, health care organizations, health insurance companies, pharmaceutical and medical products industry. Patient rights refer to a list of rights (right to privacy, right to confidentiality, right to informed consent etc.) conferred to care-seeking individuals in health care settings [2]. A well-established, legally supported, and clearly defined set of patient rights reflects core health care ethical principles such as autonomy, justice, beneficence, and non-maleficence [3]. It is also helpful to standardize health care for population and empowers care-seeking individuals to know what to expect of their health care system. Empirical studies assert that realizing patient rights is a tangible entry point to infuse accountability and enhance overall quality of health care [4–6]. Scholars and international organizations such as the World Health Organization claim that patient rights are a central element to build people-centered health systems, to ensure dignity for health care seeking individuals and to realize justice in health care provision [6–9].

A growing body of literature on patient empowerment shows that patients with accumulated disadvantages such as age, race and poverty tend to experience a feeling of powerlessness during health care encounters [10, 11]. A wide array of factors related to patient (e.g., health literacy, family pressure, state of being afflicted with a frustrating disease) provider (e.g. inexperienced provider, conflict within the treatment team), health system (e.g., resource constraints, high patient-provider ratio in emergency care settings, high cost of health care, documentation burden) and patient provider mismatches (e.g., language barriers, cultural barriers, locus of control) can contribute to a situation where the dignity and rights of patients are ignored [12]. An important United Nations’ report highlights that patients as individuals are at a vulnerable position for exploitation and degrading treatment in health care contexts [13]. Every patient is less powerful in relation to their care-provider due to their state of dependency for medical treatment and cure. The complex structuring of medical care systems together with high cost of care prove to be challenging for individuals to receive health care in alignment with human rights principles [2]. In addition, positions related to gender, race, caste, and other socio-economic factors put patients and their family members in a disadvantaged state [14–16]. Across the globe, phrases such as patient centered care, shared decision making, patient emancipation in health care are gaining momentum [17–20]. At the heart of realizing patient centered care, shared decision-making and patient emancipation in health care is to address the unjust power imbalance between care-seeking individuals and care-providers [21, 22]. In this regard, patient rights serve as a key counterforce to paternalistic approaches of health care provision and strive to move power decisively in favor of patients. Patient rights play a key role in navigating the complex health care systems and at the same time receive health services in alignment with human rights [2]. Normatively, patient rights provide the grounds for the citizens to engage and take control of health care processes they experience [23].

But in reality, both in low- and middle-income countries and in high-income countries, serious patient rights violations such as denial of health care when in need, disrespectful care and exploitations such as holding patients in hospitals when they are unable to pay health care bills happen [24–29]. Empirical studies across the contexts found that socially disadvantaged care-seeking individuals hardly claim their health care related rights [30–34]. Few studies indicate that lack of awareness on patient rights  [35–37], ineffective health care regulatory oversight and poor resources for health care provision as some factors that engender patient rights violations in health facilities [38]. We argue that challenges to implement patient rights in health facilities should also be understood in the realm of competing discourses on patient rights. Furthermore, conceptually the normative ideas on patient rights are grounded in human rights, ethical, legal, and moral principles [1–3, 9, 39]. Yet, empirical work that seeks to explore the translation of conceptual understandings of patient rights to practice in the everyday lives of health system actors and care-seeking individuals is rare. To this end, an inquiry into the discourses on patient rights will be useful to explore the gap between theory and practice.

By discourses we refer to the language that is used in “social situations and social practices” [40]. Discourses are “social spaces” where actors interact and represent the social world. The processes of interaction and representation of the social world are not neutral. These processes are imbued with power asymmetries between actors, which manifest in implicit and explicit content of varied types of text. Discourse thus not only includes written and spoken words but also a wide variety of images, sounds, gestures, and other forms of communicative action in social life [41, 42]. Put differently, the challenges to implement patient rights can be captured by critically examining how the idea of patient rights is perceived and enacted by various actors. It means examining “who is allowed to say, write, hear, read what to/from whom, where when and how” (of patient rights) [43].We maintain that patient rights is a contested terrain and is subjected to politicization due to the role of power and politics in health care provision and patient-provider relationships. In a normative sense, patient’s rights are recognized as one of the critical tools, strategies, and a means for dealing with power asymmetry in health care provision and to build national health systems that are fair, just, and sensitive to equity concerns [23]. Therefore, it is crucial to explore how various actors in health facilities and beyond, compete/co-ordinate to legitimize their interpretations and practices and attempt to promote/repress patient rights. We assert that relatively powerful actors such as health care-providers deploy a panoply of discursive resources to promote, reinforce, and sustain certain kind of discourses on patient rights.

Though there are several ways of doing a discourse analysis, we purposively chose critical discourse analysis (CDA) approach. This is because the analytic focus of the present study was to explore how actors wield their power through discourses and promote/suppress patient rights in health facilities. The basic premise of CDA is that we use language in a purposeful manner, and our discursive choices may be intentional or unintentional [43]. CDA requires an exploration of the language use of those who wield power as it contributes to the (re)production of social domination of one group over the other [44]. Critical theorists such as Fairclough (2015) [42] & Van Dijk (1988) [43] contend that a critical analysis of language provides deeper insights into ideas, practices and power relations that are often not explicit. Furthermore, Gasper (2022) [45] argues that the purpose of CDA is to challenge the power structures that embody “systems of social exclusion and injustices in the linguistic systems”. Van Dijk (2017) [46] indicates that by examining the choice of words, sequences, patterns, omissions, additions, metaphors, euphemisms, conspicuous, inconspicuous communicative actions, utterances, silences, and expressions, we will be able to understand how social-political practices (on patient rights) are discursively constructed. CDA also enables us to understand various discursive resources which are linguistic instruments that are available for certain actors and aid in constructing specific discourses, representation of social groups and shape shared meanings of social realities [47, 48].

Thus, discourse is not only about ways of thinking but also about (conscious and unconscious) acts of doing in social life. A dominant discourse plays into power dynamics to define how people should be approached, thought of, represented, and studied. Gradually through subtle and hidden ways, dominant discourses are internalized by people, become a socio-cultural reality and construct knowledge in a certain way [49]. If we pay attention to the routines and practices of text production, and the circumstances and practices through and in which the audience consume the discourse, we can understand the power dynamics [41]. Finally, a critical perspective on patient rights discourse identifies whether or not the patient rights regime is built on the notion of human rights. A critical evaluation can reveal the plurality of understanding and interpretation of patient rights.

There are two main theoretical claims in this research. 1) There are various discourses and (discursive) practices on patient rights in policies and in health facilities 2) the power dynamics between actors in the use of discourse genres, discursive events and specific contexts enable or restrain the promotion of patient rights in health facilities.

To illustrate our theoretical claims, we present the case of Karnataka, a southern state in a low-and-middle income country (LMIC), India. India had endorsed human rights framework constitutionally [50]. Nonetheless, there are several research studies and media reports from India on patient rights violations in health facilities such as abuse, disrespectful care, unethical practices by the care-providers, denial of health care, exorbitant charging of the patients by the private hospitals, discrimination of care-seeking individuals based on their caste and other socio-economic precarities [51–59]. Notwithstanding with these disturbing findings on patient rights violations, the discontent and frustration between patients, health care- providers and health care systems is expressed in the form of violence against health care-providers and facilities [60–62]. The Karnataka state is a microcosm of a pluralistic mixed health care system in India with diverse range of health care-providers. Thus, it offers an empirical site to study the power of discourses on patient rights and analytically generalize the findings to settings like India. Tables 1 and 2 present a summary of the key national and state policies relevant to Karnataka, India on patient rights.

**Table 1:** Patient rights in policies for government health facilities in Karnataka, India

There is no legislation that provides a consolidated legally recognized list of patient rights in one place. It is scattered in multiple policies such as administrative laws concerning government employees, health professional laws and other general grievance redressal related laws. The government health facilities endorsing national quality assurance programme are provided with the following list of patient rights to display in health facilities [63]. The list includes
•Right to information •Right to access care that is sensitive to gender, religious and cultural needs •Right to privacy, confidentiality, and dignity •Right to informed decision making •Right to defined framework for ethical management including dilemma confronted during delivery of services at public health facilities

**Table 2:** Patient rights in policies for private health facilities in Karnataka, India

Unlike for the government health facilities, two specific legislations clearly articulate the legally recognized list of patient rights for private health facilities in Karnataka. We provide a summary of the rights indicated in the two legislations
**The Karnataka Private Medical Establishment Act 2017 **[64]
•Right to care •Right to confidentiality and dignity •Right to information •Right to preferences •Right to redress
**The Consumer Protection Act, 2019 **[65]
•Right to be protected against the exploitation of market •Right to information •Right to consumer awareness •Right to be assured where possible goods and services at competitive prices •Right to be heard and grievance redressal
Apart from these two legislations, health care workers in private health facilities are also governed by health profession related laws that have implications on patient rights

## Methodology

Adequate access and engagement of researchers with policy makers/practitioners is key in maintaining rigour in health policy and systems research and also for improved research uptake [66]. Our previous health system related research project experiences in Mysuru facilitated access to the district health administration. Mysuru is a typical district in Karnataka state, India with more than 1500 licensed private health facilities and several government health facilities. Due to poor implementation of the Karnataka Private Medical Establishment Act (KPMEA), 2007 the exact number of private health facilities is not known [67]. Between January 2021 and December 2021, we collected qualitative data from ten health facilities in Mysuru district. The five government health facilities for this study were selected in consultation with the district health administration. Many private health facilities that we approached during the exploratory phase of the patient rights study refused to participate citing reasons such as lack of willingness and non-approval from private health facility owners and administrators. Therefore, this study leveraged on the institutional and professional networks of the authors in India to select five private health facilities in the Mysuru district.

The ten health facilities were selected to ensure case diversity with respect to the type of health care services provided (primary or secondary), ownership (public or private), setting (urban, rural, and semi urban), health care quality accreditation status of the facility and system of medicine followed (Ayurveda system of medicine or Modern system of medicine). There were four main data collection techniques (Additional file 1-Table 1): (i) interviews (78); (ii) focus group discussions (03); (iii) participant observation (04); and (iv) document reviews. The diverse data sources and methods enabled data triangulation and examination of the use of patient rights language in the policy landscape and everyday social practices at the health facilities. MP conducted interviews and where possible focus group discussions (FGDs) with purposively selected health care-providers, health facility administrators, public health officials, care-seeking individuals, and their family members (Additional file 2- Table 1 & Table 2). Interviews were held with government officials of the district health administration, Karnataka state health department, and representatives of associations of health care organizations in India. Due to COVID-19 pandemic-related restrictions, five interviews were held through internet-based audio–video conferencing or phone. In four health facilities, we conducted participant observation in select patient waiting areas, doctor consultation rooms, outpatient, and inpatient wards. The participant observation guide and the interview guides used for this study are available in Additional files 3 and 4 respectively. Each interview session ranged from 20–45 min. Most interviews and FGDs were audio recorded, when audio recording was not possible, MP took detailed notes during and following the interviews. These notes were then shared with the participants for validation. A few care-providers, administrators, government officials shared handouts, brochures, policy manuals related to patient rights during the interviews. We also included State laws and policies relevant to patient rights in the analysis (Additional file 1-Table 1). We collected data until we reached theoretical saturation. All authors are experienced qualitative researchers in the domain of health policy and health systems.

The authors discussed the emergent themes and insights periodically and determined subsequent data collection and analysis. We used NVivo 11 to organize the data and conducted a thematic analysis [68] to identify categories, themes, roles and responsibilities of actors (thereby determining their power) located within the text (oral and written) and social practices on patient rights at health facilities. We followed the COREQ guidelines to report this qualitative research [69].

## Results

We have organized the results section in three parts. First, we present the various discourses and discursive practices concerning patient rights. Secondly, we interpret the power of care-providers and health facility administrators in shaping the discourses and discursive practices on patient rights. Finally, we illustrate the outcome of the existing discourses and discursive practices on patient rights for care-seeking individuals.

### Discourses on patient rights

#### Patient rights means raising grievances

A few care-providers indicated that patient rights were all about grievance redressal:

“In case of medical negligence, patients can go for grievance redressal. This is patient right” – (KII-Ayurveda Practitioner, Member of a district regulatory body for private hospitals).

One senior health care administrator opined that patient rights discussions come to fore only during the adjudication of medical negligence cases in courts. *– (KII- Representative, Body for quality accredited health care organizations).*

Despite reducing patient rights to mere grievance redressal, the following discursive practices in health facilities deter the effective realization of right to grievance redressal by the care-seeking individuals or their families.

None of the government health facilities having rights charters had included the right to complaint and grievance redressal in their charters. This would implicitly mean that people getting free health services are not expected to raise grievances. Two government health facilities and one private health facility had mounted suggestion/complaint box on the walls inside the health facilities. At one government health facility, the complaint table was located inside the health facility administrator’s office. Such a practice displays the sheer power of the health facility administrator and might raise a question on (physical and psychological) access to grievance redressal for care-seeking individuals in that health facility.

A few health facilities had the practice of collecting feedback from patients about their health care experiences. These feedback forms are many a times filled by care -seeking individuals in the presence of a care-provider or by the care-providers themselves on behalf of the patients (data from participant observation in health facilities). Many care-seeking individuals expressed that they have hardly raised complaints or provided suggestions in the table kept in facilities due to reasons such as fear of being seen as a trouble-maker by the care-providers, anticipating negative consequences such as wrong treatment by care-providers for raising a complaint, unwillingness and a perceived feeling that their complaints/suggestions may not yield any response from health facilities.

### Patient rights is about availing benefits of specific government health care schemes

Many care-seeking individuals and care-providers reduced patient rights to availing specific government health-care related programmes.

“They (care- providers) do speak about it if there is a major operation. It’s regarding free services that can be availed using the social health insurance card” (KII-43 years old, male farmer, private health facility).

“Whatever facilities are provided by the government, patients have the right to ask about it”- (KII-Nurse, government health facility)

During participant observation, it was found that the walls of government health facilities especially in the patient waiting areas were inundated with technical information on various government funded health programmes.

### Rooting patient rights in quality of care, consumerist and economic discourses

Patient rights related texts were present within the policies concerning health care quality assurance and consumer protection (refer Tables 1 and 2). Thus, within the policy landscape, the idea of patient rights was predominantly advanced as an aspect of quality health care and consumerism. Several care-providers/administrators/officials highlighted resource constraints as a reason for their inability to promote patient rights. They indicated constraints such as health workforce shortages and poor public health infrastructure even to provide basic health care to the population. According to a medical intern:

“If he(patient) is financially not equipped, what can we do. Sometime there are government schemes… We can just play our part: explain everything, this is this… that’s it after that it’s patient choice”- (FGD1-Medical intern, government health facility).

In resource-limited government health facilities, there is a trade-off between respecting patient rights and everyday task completion. The following quote from a specialist doctor illustrates this:

“People sitting in a long queue with crying babies in hand. Mother is anxious but I cannot answer everything. There are times, I say to patients that to study this it has taken me 5 years now you are asking me to explain within 5 min. I cannot answer you. This is how also you respond, not that every time you explain everything to patient)”- (KII-Anaesthetist, government health facility).

A senior medical professional holding an administrative position in a body for quality accredited health care organizations wondered if patient rights is a luxury in India since even access to basic health care itself is a huge struggle for ordinary citizens.

*“A person in the marginalized section of society, is not even getting the basic treatment and we are talking about his rights. Rights issue cannot become more important than providing him good clinical care* *… it’s like asking for a neck tie when you don’t have underwear”*

“the state has failed to given even adequate care to the people. So once you start doing that and I will say that the state has to show the application of these rights” – (KII- Representative, Body for quality accredited health care organizations).

Concurring with the consumerist view, several care-seeking individuals and their family members opined that one should have money to enjoy all patient rights:

“Nobody speaks this (patient rights)… It’s all about money. If you pay money, they (care-providers) behave well with you”(KII- Female, 50 s, daughter of a care seeking individual, private health facility).

Another care seeking individual indicated inadequate access to basic medical care let alone claim patient rights.

“We are poor people, we should not talk about rights. We should just receive the care what they(care-providers) provide. We should not question them” (KII- Male, 63 years, care-seeking individual, government health facility).

### Framing patient rights as a contributory factor for violence against health care professionals in resource limited settings

Several care-providers propagated the economic discourse on patient rights by asserting that promoting patient rights in resource limited settings will trigger violence against care-providers and health facilities. This discourse of care-providers was bolstered through the practice of displaying posters warning care-seeking individuals against any form of violence against health care-providers and facilities. All ten health facilities had posters warning care-seeking individuals on legal action and imprisonment for violence against health care- providers. However, information on patient rights was not displayed in many facilities:

“I have seen health facilities mentioning about penalty for violence against medical professionals but not about (patient) rights”- (FGD2-Care seeking individual, private health facility).

Many care-providers spontaneously indicated increasing trend of violence against health care-providers and related it to patient rights. A doctor who had witnessed mob-attacks earlier in a government health facility feared promoting patient rights could exacerbate violence against health care-providers in resource limited settings*:*

“Even without talking about patient rights, there is increasing violence against the health care- providers if we encourage patient rights, things might become worse”- (KII-Pediatrician, government health facility).

A few nurses and a doctor felt there could be negative consequences of promoting patient rights in a context where resources are limited. They feared that patients would claim services that cannot be provided:

“I don’t think that will be useful (i.e. publishing patient rights in newspaper), people will take it in some other way. If we say that they can avail all facilities, then patient will act in some other ways and start getting adamant about those facilities. They will ask that you have given the advertisement, so what is the problem in providing the facility”- (KII- Senior nurse, government health facility).

Resources such as competent health workforce and health infrastructure are indeed essential for providing quality health care. Nevertheless, resource constraints provided an opportunity for care-providers/administrators and authorities to be less accountable and justify their practices for not promoting patient rights explicitly in policies and health facilities.

### Emphasis on patient responsibilities than patient rights

It was also noted that care-providers, health facility administrators and public officials laid emphasis on patient responsibilities than patient rights. Laying emphasis on patient responsibilities was a discourse tactic used by care-providers, health facility administrators and public officials to negate patient rights. In one government health facility where the charter was displayed, the number of items under the list of patient responsibilities was long compared to the number of items in the list of patient rights. A significant number of care-providers/ health administrators felt that patient responsibilities and duties are more important than patient rights. See for instance the following quote,

“Before talking about rights, responsibilities should be emphasized. For example, it is the right of the patient to have a clean hospital. Our staff are cleaning the hospital all the time. But the patients dirty it. They dump napkins in toilets and damage the windows. If that is the case, how can we maintain cleanliness in hospital. For every right there are responsibilities for the patient”- (KII-Administrative Medical Officer, government health facility).

Some of the patient responsibilities indicated in the charters and interviews were as follows: 1) follow the rules of hospital 2) respect the rights of care-providers 3) comply with treatment 4) provide complete personal and health related information 5) clarify doubts with care- providers 6) report any untoward reaction to the given treatment 7) accept measures taken by the hospital to ensure confidentiality and privacy of medical records 8) refrain from making unreasonable demands.

The care-seeking individuals also internalised the idea that patient responsibilities are more important than patient rights. Several care-seeking individuals and their family members expressed during interviews that they have responsibilities such as following the rules of hospital and abide by the instructions of care-providers. They also felt that questioning the care-providers is not the right thing to do since such acts will have implications on the way they get treated in the hospital. Some care-seeking individuals justified that as they were poor they had no choice but to endure disrespectful behaviour of the hospital staff. In the crowded government health facilities, it was observed that care-seeking individuals waited in serpentine queues for long hours for doctor consultation and some of them who were interviewed for this study did not complain about the long waiting time.

### Who controls the discourse on patient rights in health facilities?

To understand the discourses of care-seeking individuals and their family members we had to rely mostly on key informant interviews and FGDs whereas for care-providers and others, we had multiple data sources (policy documents, patient rights charters, health records, posters/pictures displayed in health facilities and patient feedback form templates). Thus, care-providers and others had access to more discursive resources to shape the notion of patient rights than the care-seeking individuals and their family members. The care-providers also had the discretionary power to decide the extent to which care-seeking individuals should have access to discursive resources (e.g. distribution of pamphlets on patient rights).

As care-providers and health administrators possessed more discursive resources than care-seeking individuals. It was easy for them to employ various discourse strategies to sideline patient rights discourses. They embedded patient rights within consumerist discourses using narratives such as patient rights demand (financial) resources. Subnational health care policies in Karnataka framed patient rights as an aspect of quality health care (Refer Table 3). This economic consumerist discourse was so powerful that care-seeking individuals normalized the idea that realization of patient rights is a function of financial resources. The other discourse strategy of care-providers and health facility administrators was to lay emphasis on patient responsibilities than patient rights and downplay patient rights by framing it as a matter of grievance redressal and benefitting from specific government health programmes.

**Table 3:** Framing of patient rights in subnational health care policies and in medical/nursing curriculum

Patient rights provisions were scattered across multiple subnational policies. For instance, patient rights provisions in KPMEA are mainly for private health facilities. The National Quality Assurance Programme launched in 2013 also emphasizes on patient rights but is only for public health facilities. Apart from these, health facilities can also voluntarily opt for accreditation from the National Accreditation Board for Hospitals and Health Care Providers which mandates the display of patient rights charter in health facilities. Then there is LaQshya programme striving to achieve respectful maternity care in government health facilities. The implementation of regulatory policies to improve quality of care is suboptimal. Such policies were viewed as a licensing tool rather than an instrument for implementing patient rights:
*“Those licensing requirements (KPMEA) are more on infrastructure and human resources. I don’t think that any licensing requirement is talking about patient rights”- (KII-Representative, Body for quality accredited health care organizations)*
State policies for regulating the conduct of care-providers such as the doctors and nurses do not mention the term “patient rights”. Rather in these regulations, patient rights are implicated as a matter of health professional’s obligations towards care-seeking individuals and avoiding medical malpractices (cfr Indian Medical Council (professional conduct, etiquette, and ethics) regulations, 2002; The Karnataka nurses, midwives & health visitors’ rules, 1964)
In the nursing and medical curriculum, elements of patient rights such as confidentiality, privacy and informed consent are taught from a legal perspective. The Medical Council of India, now renamed as the National Medical Commission, provided guidelines in 2018 to emphasize attitude, ethics, and communication related competencies for Indian medical graduates. However, these are only guidelines that are left entirely to the discretion of medical institutions for its implementation. The medical or the nursing curriculum do not articulate explicitly rights-based approach to health care. The health professional curriculum orients the budding graduates with the idea of rights in a piece-meal fashion (e.g., rights of psychiatric patients, child rights) (cfr undergraduate and post graduate curriculum of National Medical Commission, 2022 and Indian Nursing Council, 2022)

Passive dissemination of patient rights-related texts in health facilities disclose the relative power of care-providers and health facility administrators to determine the discursive practices on patient rights (refer Tables 3 and 4). See for instance, the practices concerning patient rights charters. Patient rights charters are one way in which discourses are codified and transmitted textually. Patient rights charters were authored mainly by health facility administrators and care-providers with little or no involvement of care-seeking individuals. Patient rights posters were displayed only in limited places. Care-providers expected the care-seeking individuals to know patient rights on their own. But care-seeking individuals expected that care-providers actively disseminate information on patient rights.

**Table 4:** Some discursive practices that suppress patient rights in health facilities

Normatively, policies designed by the State, provide frameworks, and guide implementation of patient rights at health facilities. The KPME Act, 2007 mandates the display of information regarding the charges for services provided, grievance redressal and a patient rights charter. But a majority (4/5) of the private health facilities visited during this study had not complied with this legal mandate. Of course, the display of patient rights charter alone is not sufficient to assure patient rights implementation at the health facility level. But the matter of concern is that even the minimum requirement of displaying the patient rights charter was not fulfilled by many health facilities
In three private health facilities, unqualified people were practicing as allied health professionals such as nurses, pharmacists, therapists etc. Some care-seeking individuals reported paying informal payments to care-providers in government health facilities. In a milieu of weak health care regulation, relatively powerful actors such as care- providers, public officials, health care administrators are not under sufficient pressure to comply and they tend to overlook the importance of promoting patient rights discourses. In other words, system-level efforts are not sufficient to pro-actively inform the care-seeking individuals about their rights
The standardized health records such as the Mother Card distributed to antenatal mothers in government health facilities provided only technical information about maternal and childcare but did not include information about patient rights. In one public health facility we found that the patient records had only patient responsibilities listed but not patient rights. In another public health facility in a rural setting, we found that the posters depicting the rights of women seeking maternal and childcare were in English only (i.e., in a language that is hardly understood by rural folks who are not able to read and write). Thus, we can conclude that the efforts to institutionalize patient rights at the health facility level have remained largely tokenistic

Care-providers exhibited resistance in the form of passive disregard for patient rights-related policies. For instance, all care-providers reported that patient rights were hardly found in the list of topics chosen for their in-service training. The policy frameworks concerning patient rights provided a stock of specific discursive resources to care-providers and health facility administrators. For example, they provided discretionary power to care-providers/health facility administrators to design the content of patient rights charters in health facilities. So, the charters displayed in health facilities were customized by health facilities with little or no involvement of the care-seeking individuals. Moreover, the policy frameworks acknowledged care-providers as the legitimate and credible source to produce and distribute knowledge on patient rights in health facilities. The existing discursive practices left care-seeking individuals at the mercy of care-providers and health facilities to learn about patient rights. At times even the highly educated care-seeking individuals from affluent families were uncertain about the existence of explicit list of patient rights in health facilities. Very rarely care-seeking individuals used the language of patient rights with their health care-providers. Aligning with the implicit intention of the discourses on patient rights in policies and health facilities, care-seeking individuals feared that articulating patient rights might make them appear “bad” in the eyes of their care-providers.

The care-providers especially in government health facilities expressed that promoting patient rights might trigger violence against health care-providers. But care-seeking individuals had a different view, they reported that in private hospitals, lack of transparency in the charges imposed for the treatment provided (i.e., violation of right to information) led to violence in the health facilities.

“Patients get desperate. In private hospitals they will not have money to pay bills. People would be harassed saying that only after paying money they would be given the dead body of relative. This desperation turns into violence”- (FGD 1- Male, early 50 s, care-seeking individual, Private health facility).

The fear of care-providers about violence in health care settings is reasonable because their life and well-being is at stake. But the thinking that promoting patient rights will intensify violent acts by care-seeking individuals is misconstrued by care-providers. Such a misconception is shaped in a context of limited health care resources for decent care provision. Further, in response to violence against health care professionals, stringent laws were put in place in Karnataka to punish people who assault health care-providers and cause damages to health facilities. There were posters in every health facility highlighting State laws, warning strict legal action on those who indulge in violence against health care professionals. In contrast, in most health facilities information on patient rights were not displayed. Such a differential social practice reveals the power of care-providers, health facility administrators to influence the policy and care environment where patient rights remain subdued.

The above findings suggest that health facility administrators and care-providers predominantly acted as gatekeepers on deciding what is (not) to be discoursed on patient rights in health facilities.

### Outcome of the existing discourses and discursive practices on patient rights

Existing discursive practices in health facilities have led to at least two consequences: first, care-seeking individuals hardly knew that they have rights in health facilities. Second, even if they knew, they doubted the utility of patient rights and there was a fear of reprisal for claiming patient rights. Third, care-seeking individuals and their family members had internalized some of the discourses promoted by care-providers, health facility managers, public authorities, and subnational policies such as realization of patient rights is a function of financial resources and patient responsibilities are more important than patient rights. The existing discourses and discursive practices rendered “patient rights” invisible among care-seeking individuals.

Many care-seeking individuals in health facilities even where the patient rights charters were displayed expressed their ignorance about the term patient rights or had a distorted and/or a limited understanding of patient rights. Only two of the ten health facilities had a patient rights charter available to patients through displaying it and had brochures on patient rights. However, the brochures were not distributed to all patients in the health facility. The charters on patient rights are mandatory for health facilities in Karnataka state if they are part of any quality assurance programmes. This reflects the fact that the patient rights charters and brochures are used in the health facility mainly to fulfil the requirements of health care quality assurance programmes and not necessarily in the spirit of promoting patient rights. Neither the care-providers nor the care- seeking individuals were able to list all the rights displayed in the charter of health facilities they belong to. All care-seeking individuals to whom we asked about the presence of a patient rights charter in the health facility reported that they had not paid attention to it.

“I have not heard about it. Nobody has taught about that (patient rights)”-(KII- 43 years old, male, patient, private health facility)

Some care-seeking individuals and care-providers mentioned about the limited capacity of care-seeking individuals to claim patient rights:

*“Most of them are not aware of their rights. They don’t even know that they have right to care or something like that. They’re not used to raising their voice. Most of them will be uneducated. They will be meek. They don’t know the value of their rights”- (KII-General physician, Private health facility)*

Some care-seeking individuals opined that they might be perceived as trouble makers in the eyes of care-providers or had fear to demand patient rights due to anticipated negative consequences such as retaliation and retribution from the care-providers:

*“It (patient rights) should be there and it will be there. But people are frightened, that the doctors may shout at them, hence patient don’t talk much about it”- (KII-Female, patient, a government employee, private health facility)*

One experienced care-provider acknowledged that none of her patients had used the language of rights during health care encounters and at the most they would ask additional details about the clinical treatment they receive:

“They (patients) won’t tell that word right but at the most they will ask, you are doing all this, will there be any complication”- (KII Anaesthetist, government health facility)

## Discussion

We applied CDA as a theoretical framework to explore the hidden power relations shaping the discourses on patient rights in health facilities in Karnataka. Firstly, we found that patient rights discourses are not based upon human rights notions. Discourses such as “*patient rights a luxury, patient rights are for people who have money”, realization of patient rights demands more resources* deny human rights discourses and root patient rights in economic discourses. Such a view rejects the application of human rights principles to all people without any discrimination and embeds it in a class-conscious stratified social reality.

The patient rights discourses were predominantly embedded within the logic of quality of care, economy, and consumerist perspectives. This is because patient rights discourses of the contemporary era accommodated itself to the reigning neoliberal political economy of health care [70–72]. In the neoliberalist context, market-based values such as consumerism, individual choice, economic liberalization, efficiency, and privatization thrive. Neoliberalism also diminishes the role of state in health governance, redefines care-seeking individuals as consumers and gives an upper hand to market-based governance. It paves way for weak health care regulation and resource constraints with limited state’s intervention [73]. That also means near absence of external accountability mechanisms [74], which enable the care-providers and health facility administrators to enjoy greater autonomy in deciding what should (not) be the discourse on patient rights in health facilities. In such a socio-political context, care-providers and health facility administrators enjoyed discretionary power and promoted alternative discourses such as *patient responsibilities are more important than patient rights* and *patient rights may lead to violence against health care-providers in resource limited settings. *Aligning with the neoliberal ideology [75], the patient responsibilities discourse shifts the onus of realising dignified and respectful health care onto the shoulders of care-seeking individuals and rejects the idea that the state has the obligation to protect human rights vis-à-vis patient rights. An emphasis on patient responsibilities discourse could also be attributed to the synergistic effect of neoliberal ideology on the historical position of India where rights are seen as a corollary of duties of a citizen [76]. Though India adopted human rights framework in its constitution, the historical perspective on duties could also shape the present day understanding of rights. Samuel Moyn, a legal scholar notes that human rights were firmly associated with classical liberalism until nineteenth century, it was only in twentieth century human rights were reoriented to egalitarian aspirations. However, with the rise in neoliberal ideology across globe, human rights succumbed to the political economy of the contemporary era. It started to advocate for minimum subsistence for human living rather than fighting for an “egalitarian citizenship” [77]. When the broader human rights framework has become the prisoner of neoliberalism [78], it is no wonder that patient rights- an extension of human rights to health care to lose its original aspiration of empowering patients. In so doing, it confirmed to the global pattern of neoliberalism and the same is reflected in the discourses on patient rights. Rising material inequality, economic injustices and health inequities are also attributed to neoliberal economic policies in several countries including India [79–84]. Therefore, we argue that the prevailing discourses on patient rights and its political connotations should be interpreted in the light of socio-political changes triggered and sustained by neoliberalism.

Secondly, the routines and practices concerning the development and distribution of patient rights related text (e.g. patient rights charter) in health facilities emphasized care-providers and health facility administrator’s role as controllers of discourses on patient rights and relegated care-seeking individuals to a subordinate position. Care-providers and health facility administrators used their discretionary power to determine the content of patient rights charter and decide the place and manner to display the patient rights charter in health facilities. Similarly, not investing to make patient rights related information available in the languages understandable to care-seeking individuals, and printing only patient responsibilities in medical records were subtle discursive ways of suppressing patient rights promotion in health facilities. Another discursive strategy of care-providers, health facility administrators and public authorities to silence patient rights discourses was to promote alternative discourses such as patient rights lead to violence against health care professionals in resource limited public health facilities. Silencing patient rights discourses by highlighting violence against health care professionals creates a hostile care environment. In such an environment, care-providers tend to adopt a defensive behavior from a legal point of view and care-seeking individuals might be deterred from demanding their rights because such action from care-seeking individuals can be construed as an act of violence in the eyes of care-providers. Furthermore, the near-absence of patient rights-related content in the medical/nursing curriculum and in-service education programmes for health care professionals indicate the low priority accorded to patient rights in health profession education. The use of metaphors such as “it’s like asking for a necktie when you don’t have underwear” is a discursive attempt to strip away the essentiality principle of patient rights and assign an ornamental value to patient rights in health care provision. Such discursive efforts to suppress patient rights could be driven by the perception of health care professionals that policies on patient rights promotion are threatening their professional power [85, 86]. Other possible factors that favor discursive practices suppressing patient rights include dominance of health care corporatism and austerity measures targeting public health care system because of neoliberal health policies [59, 87].

Thirdly, the subjugation of the care-seeking individuals to specific discursive practices in health facilities has led to a situation where majority of care-seeking individuals were unaware about patient rights. Instead, the understanding and actions of care-seeking individuals aligned with the dominant economic and consumerist discourses on patient rights. Social realities construct and are constructed by dominant discourses. In a care environment where patient rights promotion is made invisible in the discursive realms, a social reality of specific nature emerges. In this social reality, either patient rights related information were kept invisible to care-seeking individuals in health facilities or health facilities adopted tokenistic approaches to disseminate patient rights related information. Care-seeking individuals were discursively dissuaded to claim any rights or raise grievances against health care professionals or health facilities. For example, absence of patient rights charter in many health facilities, presence of patient complaint table in the health facility administrators office, absence of right to grievance redressal provision in the patient rights charter displayed in government health facilities indicate the discursive efforts to repress patient rights. Several studies conducted across contexts also found that care-seeking individuals lack adequate knowledge and awareness on patient rights. These studies, like ours demonstrated that care-seeking individuals relied upon the health care professionals to know about patient rights [45–49]. Even if care-seeking individuals and their families had knowledge about patient rights, most often they preferred not to claim their rights partly owing to the fear of reprisal from care-providers.

### Limitations

We did not interview care-seeking individuals who were children or receiving psychiatric treatment as that would require distinct ethical considerations and procedures. We did not inquire about patient rights specific for health research contexts. Further, the scope of this study does not include health care-providers who were considered illegal and informal according to the existing state laws. The care-seeking individuals were interviewed in health care facilities. Further, the use of audio recorders would have influenced the responses of the participants. Private health facilities included in the study were identified through the professional networks of the Indian authors. This is because despite our best efforts, randomly chosen private health facilities were not willing to participate in the study as they feared that the study had the potential to reveal patient rights violations if any in their health facility. This could have impacted the data represented in this study. The study also included the analysis of the syllabi used for health professional training of doctors and nurses. Further research is warranted in health professional curriculum planning and implementation to explore the pedagogical approaches used for patient rights education to health professionals.

## Conclusions

Our case study demonstrated that patient rights related notions are constructed and enacted discursively. Conceptually patient rights emanate from the human rights principles in health care settings. Yet, there were varied non-human rights informed discourses on patient rights at play. Because of the impact of neoliberalism, the contemporary discourses on patient rights predominantly reflected and were embedded within the consumerist, economic and quality of health care perspectives. Relatively powerful actors such as care-providers and health facility administrators used a panoply of discursive strategies such as emphasizing alternate discourses and controlling discursive resources to suppress the promotion of patient rights among care-seeking individuals in health facilities. As a result, the capacity of care-seeking individuals to know and claim patient rights was restricted. With neoliberal policies supporting austerity measures on public health care system and weak implementation of health care regulations, patient rights discourses remain subdued in health facilities.

Patient rights being conceptually entangled with human rights, it tends to reflect the political contestations of human rights such as universal vs multiple interpretations and understanding of human rights. Cultural relativists argue that human rights as a value are always defined and shaped by specific historic, geographic, socio-economic, and political contexts. In that sense, human rights cannot be premised upon empirical universalism rather it is a matter of contextual universalism [88].This calls for context specific local pluralist understanding of human rights and its various forms including patient rights [89, 90]. The empirical findings on the local expression of patient rights in the discourses allowed for theoretical insights of how conceptual understandings of patient rights translated and practiced in the everyday lives of health system actors and care-seeking individuals. Most importantly, through the critical analysis of the subnational policies and practices in health facilities, the study identified the constraints for patient rights realization in the form of discourses. Using the CDA approach we illustrated the problematic aspects of discourses and discursive practices on patient rights where health facility administrators and care-providers wielded power to oppress care-seeking individuals. From the practical point of view, the study demonstrated the limitations of care-seeking individuals in the discursive realms to assert their own agency as practitioners of (patient) rights in health facilities.

There is a need for creating disruptive dialogue spaces at multiple levels of the health care system to nurture human rights-based patient rights discourses. For example, strengthening the pedagogical approaches to teach human rights in the curriculum of health professionals is an effort in that direction. The governance challenges to promote patient rights in health facilities should consider problematizing the existing textual, oral, symbolic, and materialistic representations of patient rights in policies and in health facilities. This would help understand the subtle social practices in health facilities that disrupts the promotion of patient rights related text and talk on the ground. That way we can take steps to address them and develop an emancipatory patient rights regime.

## Supplementary Information

**Additional file 1. **Data Sources

**Additional file 2. **Socio-demographic profile

**Additional file 3. **Participant observation guide

**Additional file 4. **Topic guide-Interview

## Data Availability

The data that support the findings of this study are available from the authors. But restrictions apply to the availability of these data which were used for the current study so are not publicly available. Data are however available from the authors upon reasonable request.

## Abbreviations

CDA: Critical Discourse analysis
KPMEA: Karnataka Private Medical Establishments Act
FGD: Focus group discussion
