# Supplementary material for: “It’s like asking for a necktie when you don’t have underwear”: Discourses on patient rights in southern Karnataka, India
Source: Int J Equity Health. 2023 Mar 15;22:47. doi: 10.1186/s12939-023-01850-5 (PMC10015129; doi:10.1186/s12939-023-01850-5)
Supplement: Supplementary file 1 — Additional file 1. Data Sources [file 12939_2023_1850_MOESM1_ESM.docx]

**Additional file 1- Data sources**

**Table 1: Data sources**

| **Data sources** | **Description** | **Number** |
| --- | --- | --- |
| Interviews | Transcripts and interview notes | 78 |
| Focus group discussions | Transcripts of three FGDs held with care-seeking individuals, nurses, and medical-interns at the health facilities | 03 |
| Field notes | Thick description of the data collection experiences and observations made in health facilities | 15 |
| Document review |  |  |
| 1. Key legislations (directly and indirectly) governing patient rights in Karnataka 2. UG and PG medical/nursing curriculum proposed by National Medical Commission and Indian Nursing Council) | - Indian Medical Council (professional Conduct Etiquette and ethics) regulations, 2002 - Karnataka Private Medical Establishments Rules, 2018 - Consumer protection act, 2019 - The Karnataka nurses, midwives, and health visitors’ rules, 1964 - The Karnataka Ayurvedic, Naturopathy, Sidda, Unani & Yoga practitioners’ registration, and medical practitioners’ miscellaneous provisions act, 1961 - Since there were several post graduate(PG) courses, we chose the curriculum of two PG courses in medicine and nursing. | 11 |
| ii. Health care quality assurance programme manuals and guidelines | - National quality assurance standards for government health facilities, 2020 - National Accreditation Board for Hospitals and Healthcare Providers NABH-Standards for hospitals, 2020 | 2 |
| iii. Patient rights charters | Patient rights charter displayed on the walls of the health facilities | 2 |
| iv. Individual medical record templates | Used for patient care documentation in health facilities | 3 |
| v. Patient feedback forms | Used to collect feedback from the care-seeking individuals at the health facilities | 2 |
| vi. Pictures of the posters/signages (pertaining to patient rights) displayed in health facilities | This included the nature of information/instructions displayed on the walls of the health facilities in the patient waiting areas and other patient care areas | 50 |
