# Supplementary material for: “It’s like asking for a necktie when you don’t have underwear”: Discourses on patient rights in southern Karnataka, India
Source: Int J Equity Health. 2023 Mar 15;22:47. doi: 10.1186/s12939-023-01850-5 (PMC10015129; doi:10.1186/s12939-023-01850-5)
Supplement: Supplementary file 2 — Additional file 2. Socio-demographic profile [file 12939_2023_1850_MOESM2_ESM.docx]

**Additional file 2- Socio-demographic profile (Care-seeking individuals)**

**Table 1: Socio-demographic profile of the respondents (care-seeking individuals)**

| **Socio-demographic attributes** | **Number of respondents** |
| --- | --- |
| **Gender** |  |
| Male | 15 |
| Female | 15 |
| **Age** |  |
| 18-40 years | 16 |
| 40-60 years | 9 |
| 60 years | 5 |
| **Education** |  |
| Not able to read and write | 6 |
| Primary education | 2 |
| Secondary education | 7 |
| Higher education | 15 |
| **Residence** |  |
| Urban | 14 |
| Rural | 16 |
| **Employment status** |  |
| Working | 17 |
| Not working | 13 |
| **Category of care seeking individuals** |  |
| Inpatient | 11 |
| outpatients | 12 |
| **Family members of care seeking individuals** | 7 |

We approached selected patients and their family members above 18 years in the inpatient and outpatient areas of the health facility for this study. The patients and their family members were approached considering their availability and willingness to participate in the study. Respecting the principle of maximum variation in purposive sampling , we selected respondents in health facilities based on various socio-demographic factors, number of hospital stays, inpatient and outpatient areas and years of professional experience of the health workers

Care-seeking individuals comprised 25 % (table 2). Of the care -seeking individuals and their family members, 18% were in the age group of 18-60 years, and 19% were engaged in some form of occupation. Of the care-seeking individuals engaged in this study, 7% were not able to read and write.

**Table 2: Socio-demographic profile of the respondents- care-providers and others**

| **Socio-demographic attributes** | **Number of respondents** |
| --- | --- |
| **Category of care-providers** |  |
| Doctors | 18 |
| Nurses | 17 |
| *Other health care workers | 15 |
| **Age** |  |
| 18-40 years | 46 |
| - 1. years | 16 |
| >60 years | 3 |
| **Type of health facility** |  |
| Public | 42 |
| Private | 23 |
| **System of medicine** |  |
| Modern system of medicine | 55 |
| Ayurveda | 10 |
| **Level of health facility** |  |
| Primary | 13 |
| Secondary | 52 |
| **Locality of health facility** |  |
| Urban | 9 |
| Rural | 38 |
| Semi-urban | 18 |
| ****Other category of respondents** | 10 |

******Others category of health workers included counsellors, technicians, community outreach workers, clinical assistants, and pharmacists.*

***Other category of respondents included public health officials, health facility managers and representatives from health care organizations.*

We ensured that the routine hospital work was not interrupted due to research data collection, for example by approaching the care-providers who were less busy in their duty time. Moreover, some care-providers who were busy offered to participate in the interviews during their break time.

Most of the care-providers were doctors and nurses (38%; see table 3). More government health facilities were engaged in this study than private health facilities as most private health facilities approached declined to participate citing lack of interest in joining study on patient rights. Care-providers from modern medicine were predominant (61%). Health facilities from rural, urban, and semi-urban areas in Mysore were selected to ensure diverse socio-demographic settings.
