# Supplementary material for: “It’s like asking for a necktie when you don’t have underwear”: Discourses on patient rights in southern Karnataka, India
Source: Int J Equity Health. 2023 Mar 15;22:47. doi: 10.1186/s12939-023-01850-5 (PMC10015129; doi:10.1186/s12939-023-01850-5)
Supplement: Supplementary file 3 — Additional file 3. Participant observation guide [file 12939_2023_1850_MOESM3_ESM.docx]

**Additional file 3**

**Participant observation guide**

**Preparation for the participant observation**

Wherever possible, gather information about the observation setting apriori e.g., websites, media reports etc. Adapt the timings of the observation depending on the nature of the setting. Spend at least a minimum of three- four days in each of the selected health care facility to establish good rapport with the staff and care seeking individuals. Take all measures to make your participant observation appear natural in the setting.

**Potential areas for observation in a health facility**

Reception, patient waiting area, billing counters, pharmacy, consultation room, outpatient area, inpatient wards. In case of a big health facility, apriori select the no of inpatient and outpatient wards based on the feasibility, staff meetings, ward meetings and patient education sessions if applicable. Purposively select care seeking individuals and their family members and follow their care processes within the health facility.

**Role of the participant observer**

The researcher has dual roles to perform 1) researcher/observer 2) participant. However, the role of researcher takes precedence over the participant role. To have a conducive climate for gathering data, try to minimize any status difference between you and the respondents in the setting. If permitted, the PI/participant observer can participate in the nursing care activities. (The PI is a registered nurse and a registered midwife). Use judgement to know when to step back and facilitate some privacy for the respondents in the setting when appropriate.

**The informed consent processes**

Meet the highest authority of the health facility. Introduce yourself. Take adequate time to build rapport with the participants. Explain about the research project. If feasible, introduce the research project in staff meeting. Distribute participant information sheet to the potential participants in the observation setting. Clarify any doubts of the respondents. If it is a big organization, discuss with the appropriate authority beforehand the areas for participant observation in the health facility. Clarify your dual roles and responsibilities as a researcher and as a participant in setting. Also clarify the process of action and reporting with the respondents. Then administer informed consent separately to the highest authority of the health facility and to the other key respondents (staff, selected care seeking individuals and their family members) in the observation setting.

**Framework for observation**

| **Setting** | **Actors** | **Actors dynamics** |
| --- | --- | --- |
| Type of organization/health facility, location of the organization, neighborhood of the health facility/organization, facilities available in the setting, type of services provided, any artifacts concerning patient rights e.g. patient rights charter, leaflets, brochures, display of information on patient grievance redressal procedures, help desk for care seeking individuals, public relation office if available, access to the information materials, physical environment including the lighting, noise levels, space, seating arrangement , usual routines and practices. | Actors in the health facility- care seeking individuals, family members, health care professionals, other support staff, age, gender, type of care seeking individuals, patient volume | Observe the roles and responsibilities of various actors in the observation field. To best capture the actor dynamics, follow the processes of the action of the selected care seeking individuals and their family members within the setting. Pay attention to the provider-patient interaction, content of the communication, language, tone, gestures used by the actors, time allocated for direct patient care and simultaneous other activities of the actors, daily routine of the health care staff and the individuals under care. |

*Note: Use judgement to know when to step back and facilitate some privacy for the participant in the setting when appropriate.*

**Post observation**

Detailed notes on the observation to be written on the same day or at the most the following day of the observation. This enables the researcher to recollect the events accurately and truthfully from the observations setting.
