# Supplementary material for: “It’s like asking for a necktie when you don’t have underwear”: Discourses on patient rights in southern Karnataka, India
Source: Int J Equity Health. 2023 Mar 15;22:47. doi: 10.1186/s12939-023-01850-5 (PMC10015129; doi:10.1186/s12939-023-01850-5)
Supplement: Supplementary file 4 — Additional file 4. Topic guide-Interview [file 12939_2023_1850_MOESM4_ESM.docx]

**Additional file -4**

**Topic guide-Interviews/Focus group discussions**

*Text in Yellow – instructions for the interviewer*

This topic guide indicates the broad areas of inquiry during the interviews or Focus Group discussions. The questions neither not necessarily be posed in the sequence mentioned here nor it is mandatory to inquire all the areas mentioned here. There is a need for the interviewer to adapt the questions depending on the participant being interviewed/involved in the focus group discussion. Wherever possible study about the participant apriori through the organization’s websites or other data sources. Avoid seeking some of the already known details.

Briefly introduce yourself, explain the research project and hand over the participant information sheet and administer the informed consent. Provide adequate time for the respondent to go through the participant information sheet and the informed consent forms. Clarify any doubts asked by the respondent. If the respondent is not able to read, explain the content written in the participant information sheet and the consent form in front of a witness chosen by the respondent. Check the functioning of the audio recording equipment. Ensure comfort and privacy in the venue chosen for the interview/FGD.

| **Category of respondents** |  | **Health care providers/Health facility administrators/ Health facility managers** | |
| --- | --- | --- | --- |
|  |  | **Actions/Questions** | **Remarks** |
| **General part** |  | Can you please tell about yourself? Probes: qualification, role in the health facility,service tenure, professional experience etc |  |
| **Themes** |  |  |  |
| Knowledge on patient rights and patient grievance redressal systems |  | According to you, how a patient should be treated in a health facility? Probes: measures to protect the respect and the dignity of patients in the health facilities, forums for filing complaints, procedures to file complaints against health facility or health professionals, perception of public awareness on patient rights and grievance redressal systems |  |
| View/perception /position on patient rights and patient grievance redressal systems |  | What are your observations about the way patients are treated in health facilities? Probes: information provision, respect, dignity, quality of care, satisfaction with health care professionals/health facility, in public and private/allopathic and non- allopathic, perception of public awareness on patient rights and grievance redressal systems |  |
| Experience with/interpretation of existing patient grievance redressal systems for patient rights violations |  | What is considered as a formal and an informal complaint from the patient?  Have you come across any patient complaints on any health professionals or health facilities? How was the complaint was dealt? Probes: actors/institutions involved in dealing with the complaints, district grievance redressal committee under KPMEA, consumer protection cell, health professional council, civil and criminalcourts etc?kind of support available for health care professionals/patients receive during the patient complaint examination, any suggestions to improve the complaint procedures |  |
| Perceived power, leadership and achievement pertaining to patient rights implementation |  | How empowered are the care seeking individuals to stand for their rights in the health care processes? What makes the care seeking individuals feel empowered about their rights? What are the roles and responsibilities of the health care professionals with respect to patient rights and grievance redressal? Are there any actors who are supposed to be part of the complaint examination system but are not included presently? How can we make their voices heard in the complaint examination processes? |  |
| View about other actors and actor dynamics in the system for patient rights implementation |  | Who are all involved in resolving the patient complaints? Probes: levels (health facility, subdistrict, district) grievance redressal under KPMEA, consumer protection act, health professional conduct related acts, current procedures to file and examine the patient complaints concerning their rights violations |  |
| Challenges and facilitators for patient rights implementation |  | What helps to comply with the measures for respecting the dignity and respect for patients in health facilities? What are the challenges to practice patient rights in health facilities? Probes: challenges with different for a for patient complaint examination, suggestions to improve the procedures for filing and examining the complaints |  |
| Opinion about alternative mechanisms for grievance redressal |  | What is your opinion about some of the contentious aspects of patient complaints examination systems in Karnataka? E.g.inclusion of lay person in the grievance redressal committees, external regulation of health professions, ombudsman, Health tribunals probe: alternative ways to resolve patient complaints |  |
| **Category of respondents** |  | **Public (health) authorities/representatives from public institutions** |  |
| **General part** |  | Can you please tell about yourself? Probes: qualification, role in the health facility, service tenure, professional experience etc |  |
| **Themes** |  |  |  |
| Knowledge on patient rights and patient grievance redressal systems |  | According to you, how a patient should be treated in a health facility? Probes: current state of practice on patient rights in health facilities, any gaps identified, policies of the state that are intended to protect the rights of the patients in health facilities, procedures to file complaints, what level( health facility, subdistrict, district and state, procedure for private/public, allopathic and non-allopathic health facilities, perception of public awareness on patient rights and patient grievance redressal systems |  |
| View/perception /position on patient rights |  | What do you feel about the current implementation status of patient rights in health facilities in Karnataka? In your opinion, how effective are the current procedures to file and examine the patient complaints? Probe separately, the different methods in Karnataka e.g. Karnataka Private Medical Establishment Act, Consumer Protection Act, Health professional councils, Common law system etc |  |
| Experience with/interpretation of existing patient grievance redressal systems for patient rights violations |  | What do you feel about the way the patients are treated in health facilities? What do you feel about the current procedures to file and examine the patient complaints? Probes: KPMEA, Consumer protection act, health professional councils acts, civil and criminal courts etc, difference in the procedures for different kinds of facilities, any personal experience, kind of complaints received, Duration to solve the complaints, response of the public, health professional, health facility response to the complaint |  |
| Perceived power, leadership and achievement pertaining to patient rights implementation |  | Who are the actors involved in resolving the patient complaints concerning the public and private health facilities? Probe: under KPMEA grievance redressal, consumer protection cell, Health professional Councils etc, their roles and responsibilities, levels (health facility, subdistrict, district and state, Are there any actors who are supposed to be part of the complaint examination system but are not included presently, How can we make their voices heard in the complaint examination processes? |  |
| View about other actors and actor dynamics in the system for patient rights implementation |  | What kind of coordination is required between actors/institutions to resolve the complaints? How is the coordination now? Probes: stakeholder’s support/opposition to patient rights and patient grievance redressal related policies and laws, reasons they support/not support the patient rights/grievance redressal related policies/laws,  How do those policies affect their interest? What strategies they adopted to influence the policy processes? |  |
| Challenges and facilitators for patient rights implementation |  | What helps to comply with the measures to protect the respect and dignity of patients in the health facilities?  What are the challenges for practicing patient rights in health facilities? Probes: Challenges with different for a for patient complaint examination, suggestions to improve the procedures for filing and examining the complaints |  |
| Opinion about alternative mechanisms for grievance redressal |  | What is your opinion about some of the contentious aspects of patient complaints examination systems in Karnataka? E.g. inclusion of lay person in the grievance redressal committees, external regulation of health professions e.g. ombudsman, Health tribunals Probes: any suggestions for alternative ways to resolve the complaints |  |
| **Category of respondents** |  | **Representatives from non-Governmental**  **Organizations, Civil Society Organizations, Patient Organizations,**  **Private Hospital Associations, Health Professional Associations (allopathy and**  **non-allopathy) and individual public health activists/academicians.** | |
| **General part** |  | Can you please tell about yourself? Probes: qualification, role, vision, misison, activities of the organization etc | |
| **Themes** |  |  |  |
| Knowledge on patient rights and patient grievance redressal systems |  | According to you, how a patient should be treated in a health facility? Probes: any gaps identified, policies to protect the respect and dignity of patients in health facilities, procedures to file complaints about health professionals or health facility, levels where complaints are made- Health facility, subdistrict, district and state, difference in the procedures for public/private/allopathic and non-allopathic health facilities, perception on the public awareness on patient rights and patient grievance redressal systems |  |
| View/perception /position on patient rights and grievance redressal systems |  | Do you/your organization/network/association support patient rights and patient grievance redressal related policies and laws? Probes: levels of the system they engage, processes of engagement, reasons for supporting or not supporting/not supporting the patient rights/grievance redressal related policies/laws?  How do those policies affect your interest? |  |
| Experience with/interpretation of existing patient grievance redressal systems for patient rights violations |  | What has been your experience dealing with patient complaints? Probes: steps in dealing with patient complaints in different forums, user friendliness of these forums, kind of support the patient or the health facility or the health professional get during the complaint examination processes, (Probe separately for each forum for patient grievance redressal)  What do you feel about the functioning of the grievance redressal systems? Probe: Response of the public authorities/public/ health professionals/ health facility respond to the complaint examination processes? |  |
| Perceived power, leadership and achievement pertaining to patient rights implementation |  | Did you show any support/concerns about any of the policies concerning patient rights and grievance redressal systems?  Probes: How was it made possible? kind of resources had or mobilized for this, At what level of the system engagement (household, family, subdistrict, district, state, national or international), reason for engaging at this level, reaction of other actors and associations to their efforts, impact of other actors/institutions action on their efforts, any synergies or conflicts with those actors |  |
| View about other actors and actor dynamics in the system for patient rights implementation |  |  |  |
| Challenges and facilitators for patient rights implementation |  | What helps to comply with measures for protecting the respect and dignity of patients in health facilities? What do you think are the challenges for practicing patient rights in health facilities? Probe: gaps in the existing grievance redressal fora, resource requirements for effective functioning of these forums |  |
| Opinion about alternative mechanisms for grievance redressal |  | What is your opinion about some of the contentious aspects of patient complaints examination systems in Karnataka? E.g. inclusion of lay person in the grievance redressal committees, external regulation of health professions e.g. ombudsman, Health tribunals Probe: suggestions for alternative ways for grievance redressal/patient complaint examination |  |
| **Category of respondents** |  | **Care seeking individuals** | |
| **General part** |  | Can you please tell about yourself? Probe: urban/rural area, occupation, qualification etc |  |
| **Themes** |  |  |  |
| Knowledge on patient rights and grievance redressal systems |  | What are your expectations when you visit a health facility? How would you like to be treated in the health facility? Probes: informed/heard of patient rights when visited the health facilities , most important rights according to him or her, his/her understanding when we say confidentiality, privacy, dignity of patients while receiving care from a health facility, awareness on complaint filing procedures when rights are violated |  |
| View/perception /position on patient rights |  | What is the importance of patient rights? What will happen when rights are not respected? Probes: present way of treating patients in health facilities, any gaps identified? |  |
| Experience with/interpretation of existing patient grievance redressal systems for patient rights violations |  | What has been your experience concerning the patient rights in public/private health facilities, allopathic and non-allopathic health facilities probes: raised any complaint against a health facility/ health professional, complaint process experience, user friendliness of the processes, outcome of the complaint, challenges faced during the complaint process, kind of support available, expectations with respect to the complaint examination processes |  |
| Perceived power, leadership and achievement pertaining to patient rights implementation |  | Are you able to talk freely about your concerns with the health professionals/ health facility staff? Probes: supporting factors, challenges faced |  |
| View about other actors and actor dynamics in the system for patient rights implementation |  | Have you heard of any organizations/NGO/CSO working for patient rights? Probes: membership if any, their role, perception on the role and utility of such organizations in the protection of patient rights in health facilities. |  |
| Challenges and facilitators for patient rights implementation |  | What is helpful in treating the patients in health facility with respect and dignity? What are the challenges to respect patient rights? Probes: expectations from the health facilities and during the complaint examination processes |  |
| Opinion about alternative mechanisms for grievance redressal |  | Are there any alternative ways of dealing with patient complaints probes:opinion about health tribunals, ombudsman structures for dealing with patient complaints(If the patient is not aware, explain what it is |  |
| **Conclusion** |  | Ask the respondents, if they would like to add anything else to what has been discussed so far. Thank the respondent. Find out if they could be contacted again in case of any clarifications or for further information. Inform again that the research findings would be shared with the respondent. |  |
